# PAI-1 as a critical factor in the resolution of sepsis and acute kidney injury in old age

**DOI:** 10.3389/fcell.2023.1330433

**Published:** 2024-01-18

**Authors:** Maria E. C. Bruno, Sujata Mukherjee, Jamie L. Sturgill, Virgilius Cornea, Peng Yeh, Gregory S. Hawk, Hiroshi Saito, Marlene E. Starr

**Affiliations:** ^1^ Department of Surgery, University of Kentucky, Lexington, KY, United States; ^2^ Department of Pharmacology and Nutritional Sciences, University of Kentucky, Lexington, KY, United States; ^3^ Department of Microbiology, Immunology, and Molecular Genetics, University of Kentucky, Lexington, KY, United States; ^4^ Department of Pathology, University of Kentucky, Lexington, KY, United States; ^5^ Department of Statistics, University of Kentucky, Lexington, KY, United States; ^6^ Department of Physiology, University of Kentucky, Lexington, KY, United States; ^7^ Department of Pharmacology and Nutritional Sciences, Graduate Faculty of Nutritional Sciences, University of Kentucky, Lexington, KY, United States

**Keywords:** aging, biological variables, cecal slurry, kidney injury, PAI-1, sepsis

## Abstract

Elevated plasma levels of plasminogen activator inhibitor type 1 (PAI-1) are documented in patients with sepsis and levels positively correlate with disease severity and mortality. Our prior work demonstrated that PAI-1 in plasma is positively associated with acute kidney injury (AKI) in septic patients and mice. The objective of this study was to determine if PAI-1 is causally related to AKI and worse sepsis outcomes using a clinically-relevant and age-appropriate murine model of sepsis. Sepsis was induced by cecal slurry (CS)-injection to wild-type (WT, C57BL/6) and PAI-1 knockout (KO) mice at young (5–9 months) and old (18–22 months) age. Survival was monitored for at least 10 days or mice were euthanized for tissue collection at 24 or 48 h post-insult. Contrary to our expectation, PAI-1 KO mice at old age were significantly more sensitive to CS-induced sepsis compared to WT mice (24% vs. 65% survival, *p* = 0.0037). In comparison, loss of PAI-1 at young age had negligible effects on sepsis survival (86% vs. 88% survival, *p* = 0.8106) highlighting the importance of age as a biological variable. Injury to the kidney was the most apparent pathological consequence and occurred earlier in aged PAI-1 KO mice. Coagulation markers were unaffected by loss of PAI-1, suggesting thrombosis-independent mechanisms for PAI-1-mediated protection. In summary, although high PAI-1 levels are clinically associated with worse sepsis outcomes, loss of PAI-1 rendered mice more susceptible to kidney injury and death in a CS-induced model of sepsis using aged mice. These results implicate PAI-1 as a critical factor in the resolution of sepsis in old age.

## 1 Introduction

Sepsis is a life-threatening illness, characterized by organ dysfunction subsequent to a dysregulated host response to infection (Singer et al., 2016). It is a major burden to the healthcare system being the most common cause for admission to the intensive care unit and the most expensive condition treated in US hospitals (Liang et al., 2020; Rudd et al., 2020). No specific therapies aside from supportive measures are currently available to sepsis patients, which encompass a heterogenous patient population with multiple clinical phenotypes (Cohen et al., 2015; Scicluna et al., 2017; Seymour et al., 2019; Vignon et al., 2020). Sepsis primarily affects people at late middle age to old age (Angus et al., 2001; Gorina et al., 2005; Martin et al., 2006; Starr and Saito, 2014). The susceptibility of older patients to sepsis is thought to be due to increased exposure to invasive procedures, increased infection incidence, and number of comorbidities, in addition to natural age-related structural and functional changes to the major organs (Chronopoulos et al., 2010). While the average age of a sepsis patient is between 60 and 70 years (Martin et al., 2003; Rhee et al., 2017; Rhee et al., 2019), preclinical studies are habitually carried out using very young animals (Rittirsch et al., 2007; Starr and Saito, 2014). Knowledge of potential age-specific mechanisms leading to tissue damage and organ injury are lacking, and discovery of these is crucial to reducing sepsis-related morbidity and mortality.

Serine protease inhibitors (SERPINs) are a superfamily of proteins which regulate the many serine proteases, including several of the proteins involved in coagulation, fibrinolysis, and complement activation (Humphreys et al., 2023). Plasminogen activator inhibitor type 1 (PAI-1), one such SERPIN, plays a critical role in the fibrinolytic system by inhibiting the plasminogen activators (Iwaki et al., 2012). Many age-related conditions (i.e., arterial thrombosis, calcification, hair loss, amyloid deposition) and diseases (i.e., arteriosclerosis, hypertension, myocardial infarction, emphysema) are associated with decreased fibrinolytic activity and increased PAI-1 levels (Cesari et al., 2010; Eren et al., 2014). In sepsis, circulating levels of PAI-1 are further increased and high levels correlate with disease severity and mortality (Pralong et al., 1989; Kinasewitz et al., 2004; Madoiwa et al., 2006; Lorente et al., 2014; Hoppensteadt et al., 2015; Hoshino et al., 2017; Tipoe et al., 2018; Hoshino et al., 2020; Zwischenberger et al., 2021). As such, PAI-1 is thought to contribute to sepsis-related complications, including thrombosis, tissue ischemia, and disseminated intravascular coagulation (DIC) (Iwaki et al., 2012; Iba and Thachil, 2017). However, studies demonstrating a causal link are lacking.

We previously reported that circulating plasma levels of PAI-1 are associated with acute kidney injury (AKI) in two different cohorts of patients (surgical intra-abdominal sepsis patients and heterogeneous critically ill patients with sepsis-associated AKI) (Zwischenberger et al., 2021) and in mice with cecal-slurry induced sepsis (Bruno et al., 2022). Other murine studies have provided a mechanistic link between PAI-1, sepsis severity, and AKI; however, these have been limited to endotoxemia models with young mice (Gupta et al., 2015; Nakayama et al., 2023). The goal of this study was to determine if PAI-1 is causally related to AKI and worse sepsis outcomes using a clinically-relevant and age-appropriate murine model of sepsis.

## 2 Materials and methods

### 2.1 Animals and husbandry

Young male C57BL/6J mice were obtained from The Jackson Laboratory at 5–9 months of age (Stock 664). PAI-1 knockout (KO) mice were obtained from The Jackson Laboratory and bred in-house (Stock 2507, B6.129S2-Serpine1tm1Mlg/J). Aged male C57BL/6 mice were obtained from the National Institute on Aging at 18–22 months of age. All mice were housed in pressurized intraventilated cages and maintained in an environment under controlled temperature (21°C–23°C), humidity (30%–70%), and lighting (14 h/10 h, light/dark) with free access to drinking water and chow (Teklad Global No. 2918). All procedures were approved by the Institutional Animal Care and Use Committee at the University of Kentucky and performed in accord with the National Institutes of Health guidelines for ethical animal treatment.

### 2.2 CS-induced sepsis model

Polymicrobial sepsis was initiated by intraperitoneal (i.p.) injection of cecal slurry (CS) at a dose of 30–50 mg (in 300–500 µL). Dose used for each experiment depends on stock to stock variation along with consideration for age of mice and intended severity. CS was prepared as previously described (Starr et al., 2014) with minor refinements (Steele et al., 2017). Administration of antibiotics (imipenem; 1.5 mg/mouse, i.p.) and fluids (700 μL saline 0.9%, sub-Q) began 12 h following CS injection and continued twice daily for 5 days. This resuscitation protocol was developed to allow for the development of severe sepsis, while still allowing animals to survive long enough for organ damage to be studied over time (Steele et al., 2017). In selected experiments resuscitation began 24 h after CS-injection. The CS dose provided is expected to achieve LD100 when administered without therapeutic intervention with antibiotics and fluids. Body temperature, a parameter for sepsis severity, was measured with a rectal temperature probe and digital thermometer (DigiSense, Kent Scientific); mice that did not develop severe hypothermia (<30°C) were excluded from the study. Survival was monitored for at least 10 days. For experiments involving tissue collection, therapeutic intervention was carried out beginning 12 h after CS injection and continuing twice daily until the euthanasia timepoint. Non-sepsis control mice received vehicle injection (10% glycerol, i.p.) plus antibiotics, but did not receive saline.

### 2.3 Euthanasia and sample collection

Mice were deeply anesthetized by isoflurane inhalation (5% in oxygen, Covetrus) using an E-Z Anesthesia rodent vaporizer (E-Z systems). Laparotomy was performed and blood collected from the inferior vena cava by syringe needle with 10% sodium citrate. Blood was immediately centrifuged (2,500xg, 4°C, 15 min) to obtain plasma, which was stored at −80°C. Subsequently, the inferior vena cava was cut, and the entire vasculature was perfused with physiological saline through the cardiac ventricles, for the purpose of eliminating circulating cells. For histology, vessels adjacent to tissues were ligated to avoid perfusion. Major organs were harvested and snap frozen in liquid nitrogen for protein analyses or immersed in formalin for histological analyses. For lung histology, the lung was infused with formalin from the trachea prior to harvesting.

### 2.4 Plasma analyses

Interleukin 6 (IL-6, Invitrogen, BMS603-2), receptor for advanced glycation end products (RAGE, Invitrogen, EMSTK30), alanine transaminase (ALT activity, Abcam, ab105134) and D-dimer (Novus Biologicals, NBP3-08100) in plasma were assessed according to the manufacturer’s protocol. PAI-1 in plasma was assessed by Western blot analyses as described below.

### 2.5 Western blotting

Kidney proteins were extracted as recently described (Zwischenberger et al., 2021). Plasma proteins were used directly from obtained plasma. The protein concentration of each sample was evaluated by DC Protein Assay (BioRad) according to the manufacturer’s protocol. Proteins (10 μg per lane) were resolved by SDS-PAGE gel electrophoresis using TGX stain-free gradient (4%–15%) gels, visualized using stain-free technology (ChemiDoc MP Imaging System, BioRad) and transferred to polyvinylidene difluoride (PVDF) membranes (Trans-blot Turbo Transfer System, BioRad). The membranes were blocked for 1 h in 3% nonfat dry milk at room temperature and incubated with primary antibody in 1% nonfat dry milk overnight at 4°C. HRP-linked secondary antibody incubation (anti-rabbit IgG at 1:5000, HAF008, R&D, and anti-mouse IgG at 1:5000, sc-2005, Santa Cruz Biotechnology) was performed for 1 h at room temperature followed by chemiluminescence detection (Clarity Western ECL Substrate, BioRad). Primary antibodies were as follows: Rabbit anti-mouse PAI-1 (1:1000, ab182973, Abcam), rabbit anti-mouse NGAL (1:1000, ab63929, Abcam), and mouse anti-fibrin clone 59D8 (1:1000, MABS2155, Millipore Sigma). Densitometry analysis was performed on the resulting blots using Image Lab software and bands were normalized for total protein content of each lane.

### 2.6 RNA purification and quantitative real-time RT-PCR

Frozen tissues were homogenized with TRIzol reagent using the Qiagen Tissue Lyser LT (2 cycles of 3 min at 50 Hz), following standard protocol. Total cellular RNA was purified using PureLink™ RNA Mini Kit (Invitrogen, 12183018A), the concentration determined by reading the absorbance at 260 nm, and the 260:280 ratio used to access RNA purity (NanoDrop). Equivalent amounts of RNA were reverse transcribed into cDNA (SuperScript III First-Strand Synthesis SuperMix, Life Technologies, 11752-050). Quantitative real-time RT-PCR reactions for kidney injury molecule-1 (*Kim1*) were run on the QuantStudio 3 (Applied Biosystems), using Taqman gene expression assays (Applied Biosystems) for *Kim1* and *Hprt* (*Kim1*: Mm00506685_m1, *Hprt*: Mm03024075). *Kim1* gene expression was normalized to *Hprt* expression as an endogenous control. Relative quantity was calculated as 2^-(ΔCqKim1 - ΔCqHprt)^.

### 2.7 Blood bacteria

At 12 h after sepsis induction (before beginning therapeutic resuscitation), 10 µL blood were aseptically collected from mouse tail veins, diluted in 70 µL sterile saline, and plated on Brain Heart Infusion agar plates (BD, 211079). At 24 and 48h, 100 µL of the blood collected from the IVC were plated. Plates were incubated anaerobically (GazPack EZ anaerobe gas generating pouch system with indicator, BD 260683) for 48 h at 37°C. Bacteria colonies were counted and expressed as colony forming units per ml (CFU/mL).

### 2.8 Histology and scoring

Kidney, liver, and lung tissues were fixed in 10% formalin for 48 h followed by embedding in paraffin. 4–5 µm sections were cut, placed on glass slides and stained with hematoxylin and eosin (H&E), periodic acid-Schiff (PAS), and trichrome per standard protocol. Morphological changes were observed by a blinded pathologist and scored as described for each tissue. Kidney damage was assessed by three variables: tubular dilatation/flattening, tubular casts, and tubular degeneration/vacuolization. For each variable a score of 0 (<5% tubules affected), 1 (5%–33% of tubules affected), 2 (34%–66% of tubules affected), or 3 (>66% of tubules affected) was assigned as described (Eadon et al., 2012). A sum of the 3 variables provides the total kidney injury score (range 0–9). Liver injury was assessed based on the following histologic features: hepatocyte necrosis, sinusoidal congestion and edema, presence of lipid vacuoles, and infiltration of inflammatory cells as described (Inata et al., 2018). A score of 0 was assigned for normal findings and scores of 1, 2, 3, and 4 represent minimal (<25% liver involvement), mild (25%–50% liver involvement), significant (50%–75% liver involvement), and severe (>75% liver involvement) injury, respectively. A sum of the 4 variables provide the total liver injury score (range 0–16). Lung injury was assessed by morphological changes related to the presence of exudates, hyperemia/congestion, intra-alveolar hemorrhage/debris, cellular infiltration, and cellular hyperplasia. A score from 0 to 3 was assigned for none, mild, moderate, or severe injury, respectively as described (Hirano et al., 2015; Aziz et al., 2018) and a sum of the individual variable scores for each animal is presented as the total lung injury score (range 0–15).

### 2.9 Statistical analyses

Survival data were analyzed using Kaplan-Meier curves, with differences between WT and PAI-1 KO mice assessed using a log-rank test. For outcomes without a time component, such as plasma IL-6 levels, differences between the strains were assessed using a two-sample *t*-test. Since body temperature was measured at multiple timepoints for each mouse, a linear mixed model was used to analyze differences between the two strains over time. Likelihood ratio testing and Akaike Information Criterion (AIC) were used to select an appropriate covariance structure, and a Kenward-Roger adjustment was used to correct for negative bias in the standard errors and degrees of freedom calculations induced by small samples.

For all other quantitative outcomes, which involved a time component but did not involve repeated measurements from the same mouse, a full-factorial two-way ANOVA model or Poisson regression model, as appropriate, was fit to analyze differences between WT and KO mice over time. If the interaction effect between strain and time was significant, relevant pairwise differences were calculated using Fisher’s Least Significant Differences (LSD). If the interaction effect was not significant but a main effect of time was significant, separate one-way ANOVA models were fit for each strain, analyzing the effect of time after cecal slurry injection on the outcome in a scientifically meaningful way. Relevant pairwise comparisons were again performed using Fisher’s LSD when the overall time effect was significant, as appropriate.

For each model described above, a combination of visual plots and formal testing was used to evaluate all model assumptions. Log transformation of the outcome variable was used in several cases to correct for right skewness, as appropriate. Across all analyses, a *p*-value less than 0.05 was considered significant. All analyses were completed in R 4.2.1 (R Foundation for Statistical Computing; Vienna, Austria) and SAS 9.4 (SAS Institute Inc.; Cary, NC, United States).

## 3 Results

### 3.1 Lack of PAI-1 at old age renders mice more sensitive to sepsis

**FIGURE 1:**
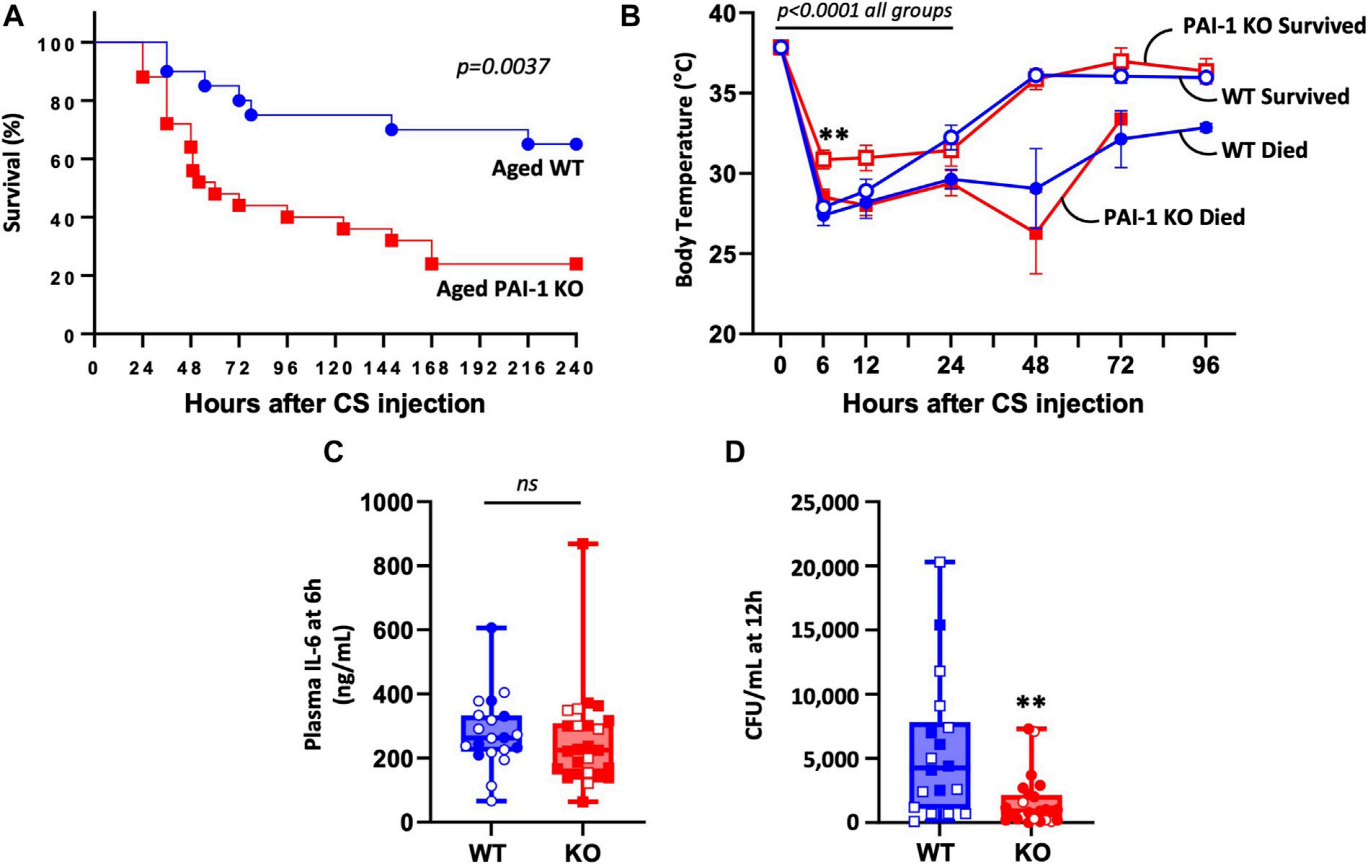
PAI-1 KO mice at old age are significantly more sensitive to sepsis compared to WT mice. Sepsis was induced in aged (20–22 months old) WT (n = 20) and PAI-1 KO (n = 25) mice by cecal slurry (CS) injection. Antibiotics and saline were administered twice daily for 5 days beginning 12 h after septic insult. Data are the combined results from two separate experiments. **(A)** Survival was monitored for at least 10 days and significance between the Kaplan-Meier survival curves was analyzed by a log rank test. **(B)** Body temperature was assessed by rectal temperature probe and curves analyzed for significance using a mixed model accounting for repeated measures while comparing the two strains and their survival status. Data are expressed as the mean ± SEM. **(C)** Plasma IL-6 levels assessed by ELISA at 6 h post CS-injection. **(D)** Bacteremia assessed by anaerobic blood culture at 12 h post CS-injection. Data are expressed in box plots from minimum to maximum values with a bar representing the mean. Open symbols represent survivors and closed symbols represent non-survivors. ** indicates *p* < 0.01 for WT vs. KO.

To determine if loss of PAI-1 protects aged mice from sepsis, we induced severe polymicrobial abdominal sepsis via cecal slurry (CS) injection. Antibiotics and fluids were provided as standard of care, twice daily for 5 days beginning at 12 h after insult. Contrary to our expectation, aged PAI-1 KO mice were significantly more susceptible to CS-induced sepsis compared to age-matched WT control mice (Figure 1A, 24% survival in KO vs. 65% survival in WT at day 10, *p* = 0.0037). Initial hypothermia was pronounced in all mice up to 24 h post CS-injection (*p* < 0.0001 compared to baseline for all group); however, after 24 h survivors were clearly distinguished from non-survivors, independent of strain (Figure 1B). While no significant difference was observed in body temperature at any timepoint between WT and KO mice that died, PAI-1 KO survivors had significantly higher body temperatures than WT survivors at 6 h (*p* = 0.0003) which was modestly sustained through 12 h (*p* = 0.0943). Despite exacerbated mortality in the KO mice, 6 h plasma IL-6 levels (Figure 1C, *p* = 0.410), often noted to correlate with mortality, were not significantly different between the strains. Interestingly, bacteremia at 12 h (prior to initiation of antibiotic therapy) was significantly reduced in the KO mice (Figure 1D, *p* = 0.003). However, additional data in separate cohorts of mice showed that this trend was not sustained, as bacteremia at 24 and 48 h post CS-injection with antibiotic treatment were similar in the two strains (Supplementary Figure S1, *p* = 0.315).

### 3.2 Young mice with CS-induced sepsis are not similarly affected by loss of PAI-1

**FIGURE 2:**
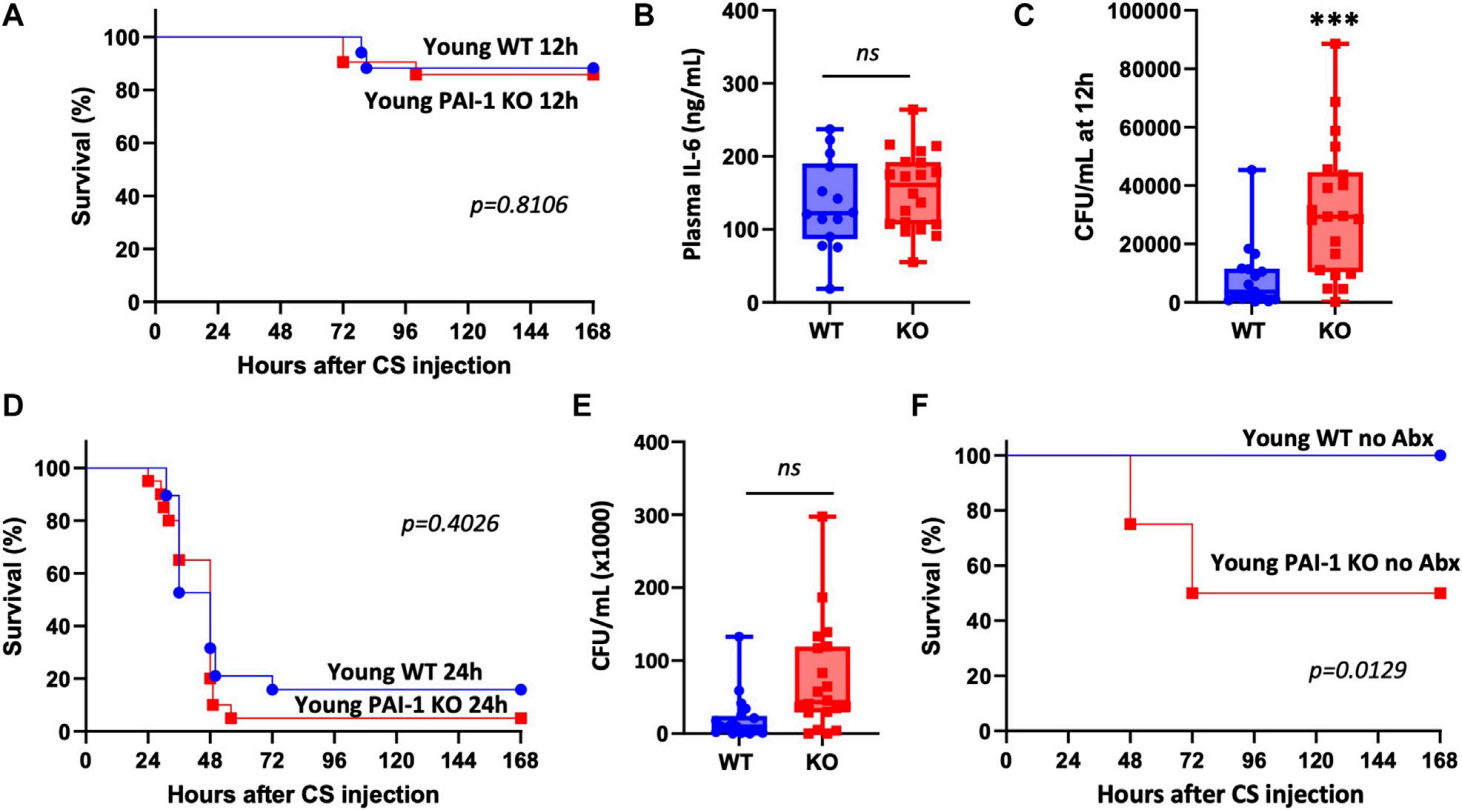
Loss of PAI-1 at young age has negligible effects on sepsis pathophysiology. **(A–C)** Sepsis was induced in young (5–9 months old) WT (n = 17) and PAI-1 KO (n = 21) mice by cecal slurry (CS) injection. Antibiotics and saline were administered twice daily for 5 days beginning 12 h after septic insult. Data are collective results from two separate experiments. **(A)** Survival was monitored for at least 10 days; log rank test was performed. **(B)** Plasma IL-6 levels assessed by ELISA at 6 h post CS-injection. **(C)** Bacteremia assessed by anaerobic blood culture at 12 h post CS-injection. Data are expressed in box plots from minimum to maximum values with a bar representing the mean, each point represents an individual mouse. Statistical difference was determined by two-sample *t*-test, ***p < 0.001. **(D)** Survival was assessed in a separate cohort of mice given a more severe septic insult by delaying antibiotic and fluid resuscitation until 24 h; WT (n = 19) and PAI-1 KO (n = 20); log rank test was performed. Data are the combined results from two separate experiments. **(E)** Bacteremia assessed by anaerobic blood culture at 12 h post CS-injection in mice from panel D. **(F)** Survival was assessed in a separate cohort of mice without providing antibiotics; WT (n = 10) and PAI-1 KO (n = 8); log rank test was performed.

To confirm whether the survival benefit with PAI-1 expression was age-specific, we conducted similar studies in young mice. There was no difference in survival after CS-induced sepsis in young WT vs. PAI-1 KO mice (Figure 2A, 87% survival in KO vs. 88% survival in WT at day 10, *p* = 0.8106). Six-hour plasma IL-6 levels were also not different between the strains (Figure 2B, *p* = 0.285); however, a significant increase in bacterial load was observed in the KO mice (Figure 2C, *p* = 0.0007). Since the induced septic-condition was mild with little mortality (possibly masking a strain-specific effect), we repeated the study, delaying antibiotic and fluid resuscitation until 24 h in order to induce more severe sepsis. Mortality was significantly increased compared to mice resuscitated at 12 h with median survival time of 48 h in both groups; however, a significant difference between the strains was still not observed (Figure 2D, 5% survival in KO vs. 16% survival in WT at day 10, *p* = 0.4026). Similar to the initial experiment with mild sepsis, bacterial load was enhanced in the KO mice; however, not reaching significance (Figure 2E, *p* = 0.163). To assess whether antibiotic administration was preferentially protecting young PAI-1 KO mice, we performed an additional survival test in young mice without antibiotic treatment. In this case, survival was significantly worse in the PAI-1 KO strain with a median survival time of 72 h (Figure 2F, 50% survival in KO vs. 100% survival in WT, *p* = 0.0129).

**FIGURE 3:**
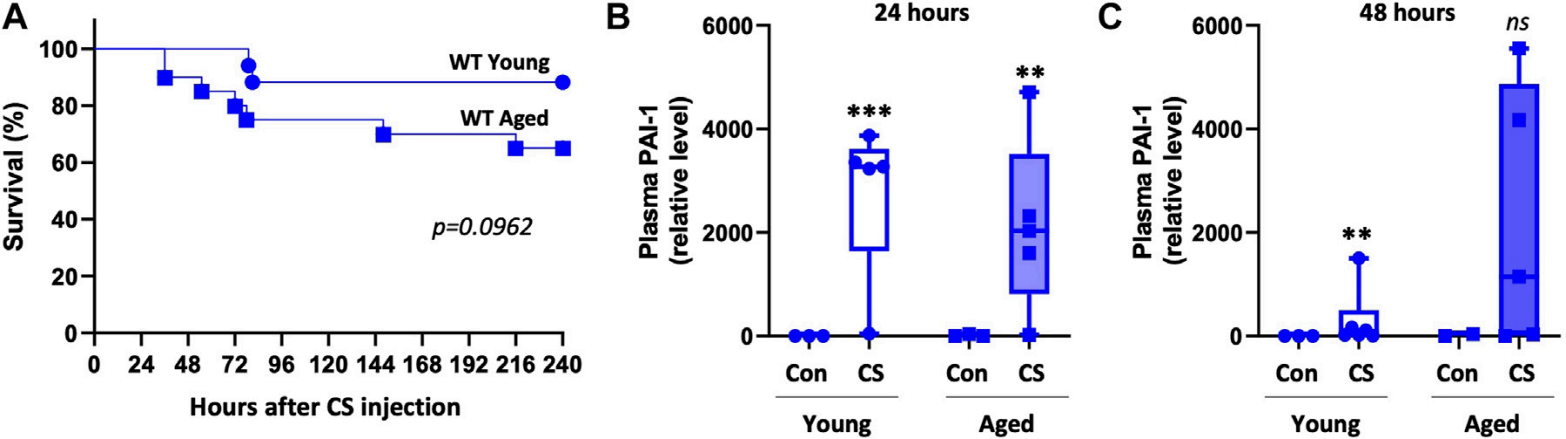
Plasma PAI-1 levels are not significantly different between young and aged WT mice. Sepsis survival and plasma PAI-1 levels were compared between young and aged WT mice from experiments already presented independently in Figures 1, 2. **(A)** Survival of young (5–9 months old, n = 17) and aged (20–22 months old, n = 20) WT mice. Significance between the Kaplan-Meier survival curves was analyzed by a log rank test. Plasma PAI-1 levels at **(B)** 24 h and **(C)** 48 h post CS-injection in a separate set of animals that were euthanized at the designated timepoints. PAI-1 was assessed by Western blot and intensity of each band was quantified after adjustment for total protein content of each lane. Data are expressed in box plots from minimum to maximum values with a bar representing the mean, statistical differences were determined by two-way ANOVA with multiple comparisons. **p < 0.01 or ***p < 0.001 compares timepoint vs. control within each age.

We then wondered whether the degree of PAI-1 upregulation in WT mice differed by aging which could explain why knockout of PAI-1 caused variable effects in young compared to aged mice. Although the experiments were done independently, survival was comparable in the young and aged WT mice that were used for the experiments described in Figures 1, 2 (Figure 3A, 88% survival in young vs. 66% survival in aged, *p* = 0.0962). Accordingly, plasma PAI-1 levels were elevated by CS-injection, but were not significantly different between WT young and WT aged mice at either timepoint (Figures 3B, C; Supplementary Figure S2, *p* = 0.988 and 0.227 for 24 h and 48h, respectively). These data suggest that differences in the level of PAI-1 between young and aged WT septic mice are not responsible for the age-dependent mortality observed with PAI-1 deletion.

### 3.3 Kidney injury is accelerated in aged PAI-1 KO mice

**FIGURE 4:**
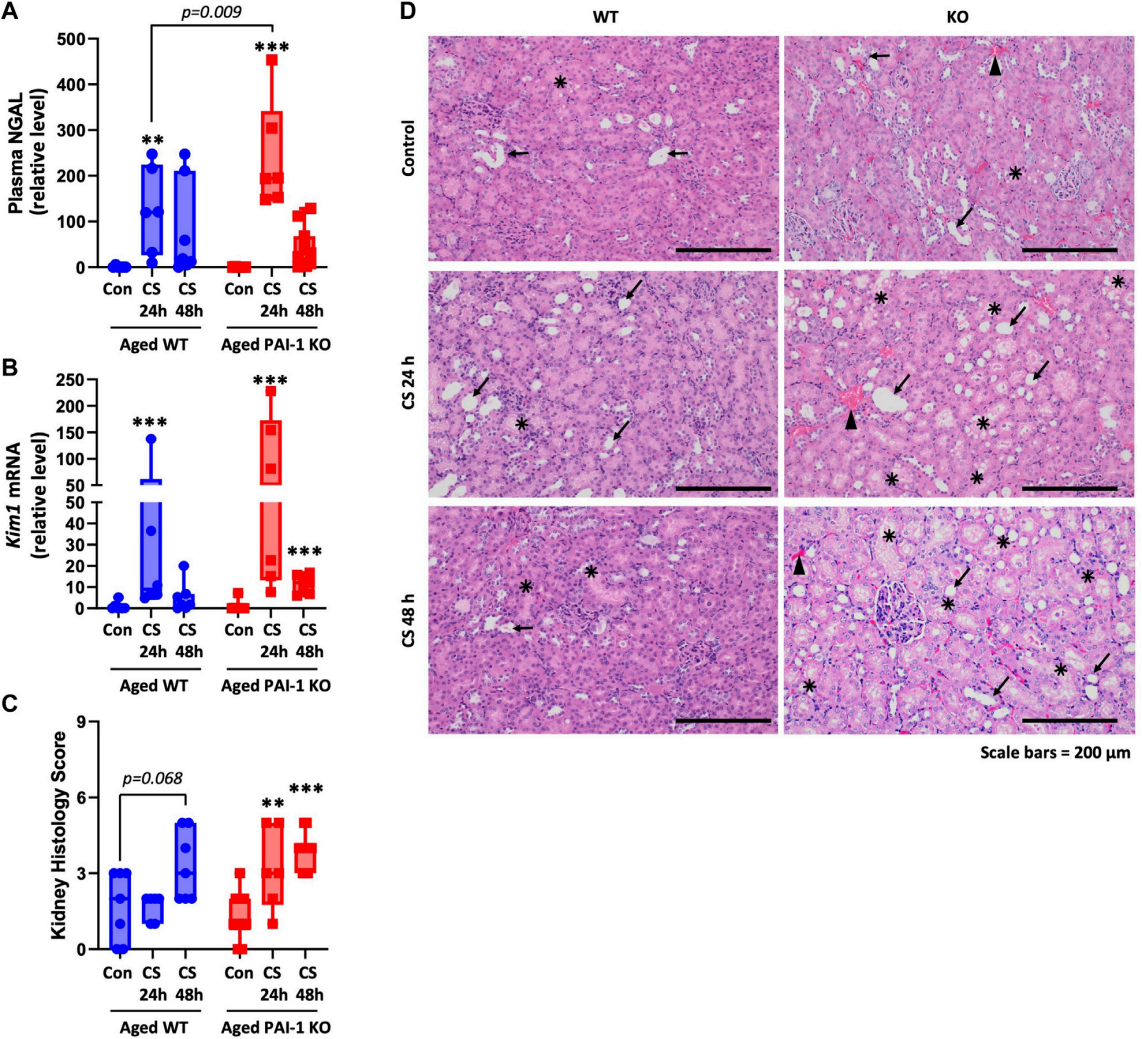
Measures of kidney injury in aged WT and PAI-1 KO mice during cecal slurry-induced sepsis. Sepsis was induced in aged (18–22 months old) WT (n = 13) and PAI-1 KO (n = 17) mice by cecal slurry (CS) injection. Glycerol-injected mice were used as controls (n = 7 WT and n = 10 PAI-1 KO). Antibiotics and saline were administered twice daily beginning 12 h after septic insult until euthanasia. Kidney injury was assessed by measuring **(A)** Plasma biomarker NGAL, **(B)** *Kim1* gene expression in kidney, and **(C)** Histological scoring of FFPE kidney tissue sections. For histological assessments, a score ranging from 0 to 3 was assigned for each of the three assessed variables and a sum of the score provides the total kidney injury score in each animal (maximum score 9). Data are expressed in box plots from minimum to maximum values with a bar representing the mean, each point represents an individual mouse. For NGAL and *Kim1*, statistical differences were determined by two-way ANOVA with multiple comparisons, **p < 0.01 or ***p < 0.001 compares timepoint vs. control within each strain. For histology, statistical differences were determined by Poisson regression, with separate models for the effect of time within each strain, **p < 0.01 or ***p < 0.001 compares timepoint vs. control within each strain. **(D)** Representative H&E-stained kidney sections (×10 total magnification); arrow indicates tubular dilatation/flattening; star mark indicates tubular degeneration/vacuolization; arrowhead indicates hyperemia.

Kidney injury was assessed using the plasma biomarker (neutrophil gelatinase-associated lipocalin) NGAL (Figure 4A; Supplementary Figure S3), *Kim1* (kidney injury molecule-1) gene expression in the kidney (Figure 4B), and by scoring of formalin-fixed paraffin-embedded (FFPE) tissue sections (Figures 4C, D). In aged WT mice, plasma NGAL levels and *Kim1* mRNA expression in the kidney were significantly increased 24 h after CS-injection (*p* = 0.006 and 0.0005, respectively) and remained elevated through 48 h although significance was lost (*p* = 0.064 and 0.063, respectively). In aged PAI-1 KO mice, plasma NGAL levels were significantly elevated at 24 h after CS-injection compared to control (*p* < 0.0001) also losing significance at 48 h (*p* = 0.241). Renal *Kim1* expression, however was significantly elevated at both timepoints in PAI-1 KO mice (*p* < 0.0001 for both 24h and 48 h compared to control). The 24 h plasma levels of NGAL were significantly higher in PAI-1 KO compared to WT mice (*p* = 0.009) and 48 h renal *Kim1* expression was moderately higher in PAI-1 KO mice (*p* = 0.074). Of note, 24h and 48 h levels were measured in different animals at euthanasia (i.e., this is not a repeated measure). Histologically, kidney injury was characterized by tubular dilatation/flattening and tubular degeneration/vacuolization (Figure 4D). Tubular casts were seen only rarely (Supplementary Figure S4), while mild focal chronic inflammation was observed in many animals, both glycerol- and CS-injected mice, likely due to the age of animals. A composite kidney injury score was assigned by summing the scores for each variable for each animal (maximum score is 9). Aged WT mice showed histological evidence of AKI only at 48 h (92% increase from control) which did not reach significance (*p* = 0.068), while aged PAI-1 KO mice showed earlier and significant evidence of AKI at 24 h (144% increase, *p* = 0.013), which was sustained through 48 h (201% increase, *p* = 0.0005). Despite the trend for worse kidney injury in the KOs, there was not a statistically significant effect of strain on mean histology score (*p* = 0.308). The survival curve of aged mice (Figure 1A) shows that most KO mice die before 48h; therefore, it is important to recognize that the data obtained at 48 h favor mice that are more likely to survive. That is, the mice with the most severe injury were represented at 24h, but not at 48 h as they were unable to survive until the tissues could be harvested for analyses.

### 3.4 Injury to liver and lung are minimal and not significantly affected by PAI-1 in aged mice

**FIGURE 5:**
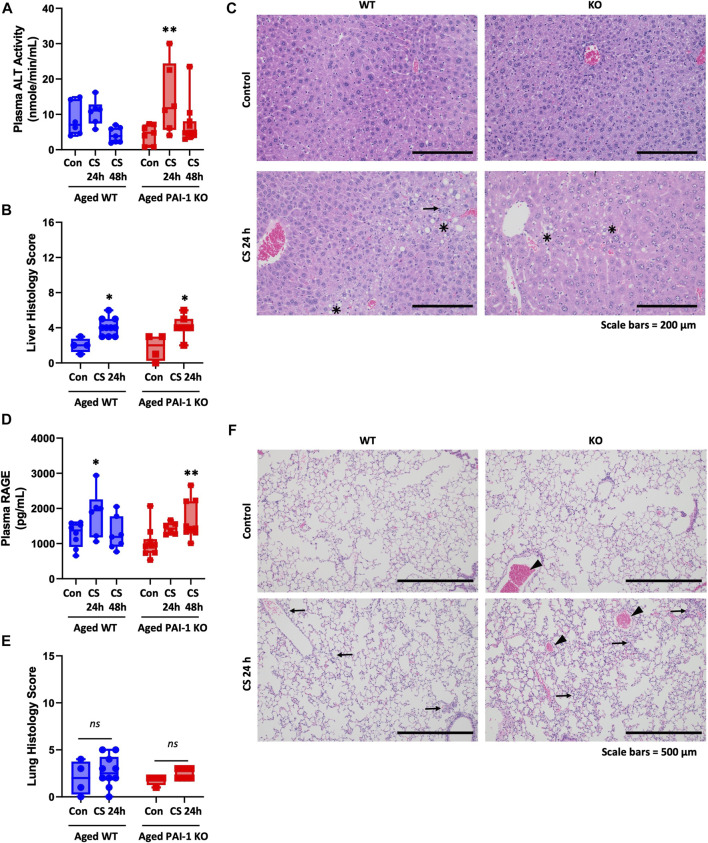
Measures of liver and lung injury in aged WT and PAI-1 KO mice during cecal slurry-induced sepsis. Sepsis was induced in aged (18–22 months old) WT (n = 13) and PAI-1 KO (n = 17) mice by cecal slurry (CS) injection. Glycerol-injected mice were used as controls (n = 7 WT and n = 10 PAI-1 KO). Antibiotics and saline were administered twice daily beginning 12 h after septic insult until euthanasia. Liver injury was assessed by measuring **(A)** Plasma ALT activity and **(B)** Histological scoring of H&E stained FFPE liver tissue sections. Liver histology score represents the sum of scores 0-4 for each of 4 measured variables for each animal, with a maximum score of 16. **(C)** Representative H&E-stained liver sections (×10 total magnification); star mark indicates lipid vacuoles, arrow indicates cellular infiltrates. Lung injury was assessed by measuring **(D)** Plasma biomarker RAGE and **(E)** Histological scoring of H&E-stained FFPE lung sections. Lung histology score represents the sum of scores 0-3 for 5 measured variables for each animal with a maximum total score of 15. **(F)** Representative H&E-stained lung sections (×5 total magnification); arrow indicates cellular infiltrates, arrowhead indicates hyperemia. Data are expressed in box plots from minimum to maximum values with a bar representing the mean, each point represents an individual mouse. For plasma biomarkers, statistical differences were determined by two-way ANOVA with multiple comparisons. For histology scores, statistical differences were determined by Poisson regression. *p < 0.05 or **p < 0.01 compares timepoint vs. control within each strain. There were no strain-dependent differences.

The liver injury biomarker alanine transaminase (ALT) activity was significantly increased at 24 h (*p* = 0.012) in the KO mice only; however, the difference between strains was not statistically significant (*p* = 0.575) (Figure 5A). Histologically, evidence of mild liver injury characterized predominantly by minimal to mild level of sinusoidal congestion, minimal to moderate steatosis (lipid vacuoles) and minimal to moderate inflammatory cell infiltration was observed in both WT and KO mice 24 h after CS-injection without significant difference between the strains (Figures 5B, C, *p* = 0.045 for WT 24 h vs. control and *p* = 0.027 for KO 24 h vs. control, *p* = 0.943 represents the strain difference). Hepatocyte necrosis was observed only in a few cases and was not dependent on strain. Overall, CS-induced liver injury was minimal with average scores of 4.10 ± 0.99 for WT and 4.14 ± 1.21 for KO (max score is 16 based on sum of four measured parameters). Minimal injury (score of 2.00 ± 1.00 for WT and 1.75 ± 1.50 for KO) was also observed in glycerol-injected control mice, likely due to natural age-related liver changes. Hydropic cell degeneration was a common observation in both glycerol- and CS-injected mice.

Plasma levels of receptor for advanced glycation end products (RAGE) were used as a biomarker for lung injury (Uchida et al., 2006; Griffiths and McAuley, 2008; Su et al., 2009) (Figure 5D). Both WT and PAI-1 KO mice showed elevated plasma RAGE levels; WT was significantly elevated only at 24 h compared to control (*p* = 0.040), while KO was modestly elevated at 24 h (*p* = 0.074, n.s.) and significantly elevated at 48 h (*p* = 0.003). There was not a statistically significant difference in plasma RAGE between the strains (*p* = 0.620). Significant histological evidence of sepsis-induced lung injury was absent in this model (Figures 5E, F, *p* = 0.286). Mild to moderate hyperemia/congestion and cellular infiltration were the most notable findings; however, they did not significantly differ between glycerol- and CS-injected mice. Exudates and intra-alveolar hemorrhage were evident only in a few cases while cellular hyperplasia was not observed in any animal.

### 3.5 Lack of PAI-1 does not affect thrombosis or fibrinolysis in aged mice with CS-induced sepsis

**FIGURE 6:**
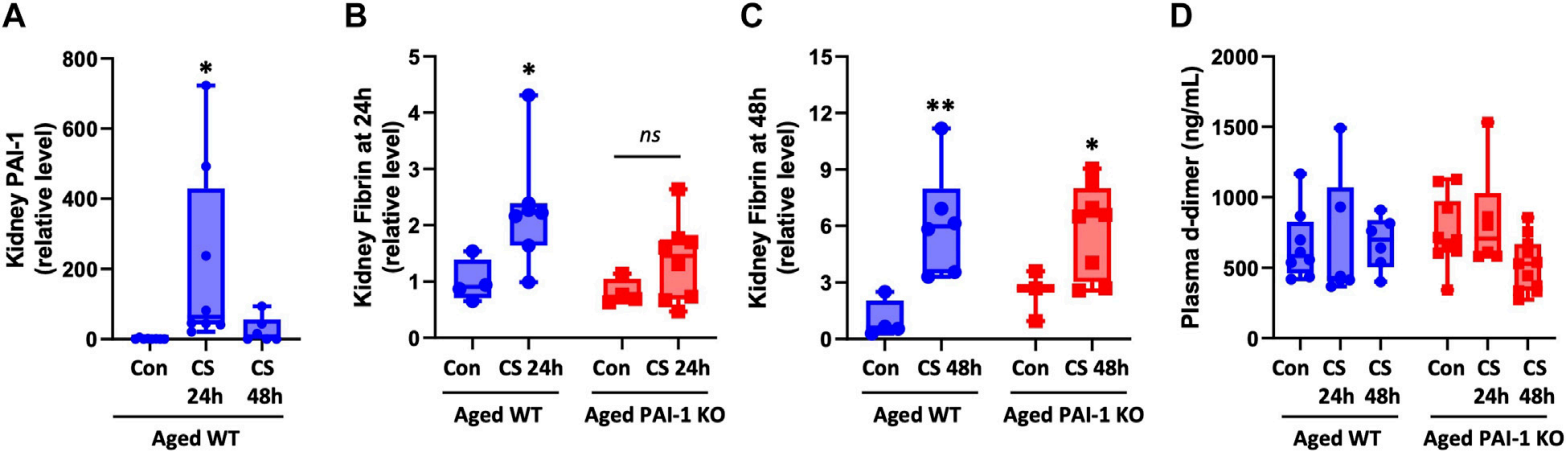
Assessment of coagulation variables in aged WT and PAI-1 KO mice during cecal slurry-induced sepsis. Sepsis was induced in aged (18–22 months old) WT (n = 13) and PAI-1 KO (n = 17) mice by cecal slurry (CS) injection. Glycerol-injected mice were used as controls (n = 7 WT and n = 10 PAI-1 KO). Antibiotics and saline were administered twice daily beginning 12 h after septic insult until euthanasia at 24 or 48 h. **(A)** Kidney PAI-1 protein levels in WT mice, *p < 0.05 compared to control. Fibrin deposition in the kidney was assessed at **(B)** 24 h and **(C)** 48 h by Western blot and intensity of each band was quantified after adjustment for total protein content of each lane. **(D)** Plasma d-dimer levels were assessed by colorimetric assay. Data are expressed in box plots from minimum to maximum values with a bar representing the mean; statistical differences were determined by one- or two-way ANOVA with multiple comparisons. *p < 0.05 and **p < 0.01 compared to control within the same strain.

Since PAI-1’s major biological role is to inhibit fibrinolysis, we assessed whether differences in fibrinolysis could account for exacerbated kidney injury in the KO animals. In WT mice, kidney PAI-1 levels were significantly increased at 24 h (*p* < 0.0001) but lost significance at 48 h (*p* = 0.089, Figure 6A and Supplementary Figure S5A); lack of PAI-1 was confirmed in KO mice (data not shown). This trend follows plasma PAI-1 levels (Figure 3; Supplementary Figure S6). Increased fibrin was detected in the kidneys of mice with sepsis at 24 and 48 h after CS injection irrespective of strain (Figures 6B, C; Supplementary Figures S5B, C). Sepsis-induced fibrin levels were not significantly different between WT and PAI-1 KO mice (*p* = 0.062 for 24 h and *p* = 0.794 for 48 h). Further, plasma d-dimer levels did not significantly differ by CS-injection or between strains (Figure 6D). Collectively, these data indicate that sepsis severity and injury to the kidney are independent of degree of thrombosis and fibrinolysis in aged mice.

## 4 Discussion

In this study using aged mice, we report that PAI-1 is protective in the setting of intra-abdominal sepsis induced by cecal slurry injection. This finding was unexpected and the mechanism of protection remains unclear, but does not appear to be mediated by PAI-1’s traditional role in inhibiting fibrinolysis. Further, lack of PAI-1 was particularly deleterious in aged mice, but had little effect on young mice highlighting the importance of age as a biological variable in studies with rodents. Most prior studies reporting a deleterious role for PAI-1 were conducted on young mice using a non-infectious endotoxemia model (Murakami et al., 1997; Gupta et al., 2015; Nakayama et al., 2023).

Although PAI-1 mediated protection was unexpected based on our hypothesis and evaluation of the literature, protective functions of PAI-1 in bacterial infections have been previously reported (Renckens et al., 2007; Goolaerts et al., 2011; Kager et al., 2011; Lim et al., 2011; Luo et al., 2011). This protection appears to be mostly related to a role for PAI-1 in bacterial clearance and neutrophil trafficking. In two different models of Gram-negative pneumonia, PAI-1 deficiency either by genetic deletion or pharmacological inhibition caused a decrease in neutrophil recruitment to the lung and increased bacterial load (Renckens et al., 2007; Goolaerts et al., 2011). In a model of melioidosis (Gram-negative), PAI-1 deficiency similarly led to enhanced bacterial load in lungs, liver, and blood which was associated with reduced survival (Kager et al., 2011). In reverse, PAI-1 overexpression improved host defense and promoted neutrophil recruitment (Renckens et al., 2007). However, beneficial effects of PAI-1 were not recapitulated in a Gram-positive model of pneumonia indicating potential pathogen-specific effects (Rijneveld et al., 2003). In our study we likewise noted that the presence of PAI-1 in young mice led to reduced bacterial load after septic insult with CS. Despite the significant difference in bacterial load, survival in young mice was unaffected. This is likely due to the fact that our model included antibiotic administration (beginning at 12 h, subsequent to initial bacterial load analyses). To verify this, we induced sepsis in a set of young mice without providing antibiotic therapy and indeed found that survival in the KO strain was significantly decreased compared to WT mice. Another prior study which used antibiotic therapy in a CLP-sepsis model also found no benefit of PAI-1 inhibition or overexpression in young mice (Raeven et al., 2014). This finding is of importance as it demonstrates the need for translational studies of sepsis to include antibiotic therapy which is provided as standard of care to patients. As such, while biologically interesting, PAI-1’s effect on bacterial clearance may be of little importance to the sepsis patient population in the clinic receiving antibiotics. Curiously, we did not observe increased bacterial load in the circulation of aged PAI-1 KO mice compared to age-matched WT, which suggests that PAI-1’s function regarding bacterial clearance may be hindered in old age.

Kidney injury was the most pronounced observation in our study. While injury to the kidney occurred in both strains, it was evident earlier (within 24 h of sepsis induction) in the PAI-1 KO animals. Plasma NGAL levels, as a surrogate for kidney damage, were increased in both WT and KO mice and the increase appeared to precede histological evidence of damage which is consistent with the literature (Haase et al., 2011). Overall level of damage, however, appeared similar between the strains. Histologically, vacuolization of tubular cells was the predominant observation which is also in line with the literature assessing the histopathology of septic acute kidney injury in rodents and postmortem human biopsies (Kosaka et al., 2016). There is evidence that morphological kidney damage does not necessarily correlate with degree of functional injury (Zarbock et al., 2014), thus it is difficult to ascertain to what degree the kidney was functionally impaired in these animals and what effect that had on survival. Recovery from tubular injury in sepsis-associated AKI is possible, thus it is tempting to hypothesize that PAI-1 plays a role in recovery from AKI leading to improved survival in WT mice compared to KO.

Prior work emphasizing a deleterious role for PAI-1 in AKI showed that young mice lacking PAI-1 have increased survival, improved kidney function, decreased apoptosis, reduced inflammation, attenuated fibrin deposition in the kidneys, and increased levels of circulating activated protein C as compared to WT mice after LPS challenge (Gupta et al., 2015; Nakayama et al., 2023). These benefits were similarly observed in a mutant mouse model where the interaction between PAI-1 and vitronectin was abolished, verifying a key role for this interaction in mediating PAI-1’s effects on the kidney (Gupta et al., 2015). In contrast, there was no benefit of PAI-1 inhibition in mice with SARS-CoV-2 infection (Nakayama et al., 2023). This could indicate that the beneficial effects of reducing PAI-1 are an LPS-specific phenomenon and occur in the absence of true infection. Indeed, deletion of PAI-1 in alveolar macrophages downregulates TLR4 mRNA and protein expression (Ren et al., 2015) and TLR4 inhibition has been shown to block PAI-1-mediated macrophage activation *in vitro* (Gupta et al., 2016). This raises the hypothesis that PAI-1 KO mice may have defective TLR4 signaling which is sufficient to protect them from LPS-induced inflammation, but not a polymicrobial infection.

PAI-1 is thought to contribute to organ injury in sepsis through its traditional role as an inhibitor of fibrinolysis thus promoting thrombosis and DIC (Madoiwa et al., 2006). This is particularly relevant for the kidney, since 70% of patients with DIC have microthrombi in the kidney at autopsy compared to 40% in the heart and liver (Levi et al., 2012). Work from our group previously showed age-dependent fibrin formation in the lung and kidney in association with high d-dimer and PAI-1 levels in a CLP-sepsis model (Starr et al., 2015). Although we expected PAI-1 KO mice to have improved kidney function due to the anticipated decrease in DIC, swinging the pendulum in the opposite direction (coagulopathy) could also be detrimental. Nevertheless, we did not observe differences in fibrin formation or d-dimer between septic WT and PAI-1 KO mice, suggesting that exacerbated mortality and AKI in aged PAI-KO mice are independent of thrombosis and fibrinolysis.

The use of aged mice in our study resulted in data contrary to the expectation regarding PAI-1 in sepsis. This expectation was based on prior studies utilizing juvenile rodents and models lacking a true infectious source, along with clinical association data. Our results highlight the importance of modeling conditions in murine studies. However, the current study does not go without its own set of limitations. PAI-1 KO mice reportedly develop spontaneous cardiac fibrosis with age (Moriwaki et al., 2004; Weisberg et al., 2005; Ghosh et al., 2010). This could underlie their susceptibility to sepsis compared to age-matched WT mice, a factor not likely to be common among the entirety of the sepsis patient population. Pre-existing cardiac fibrosis has not been noted as a major factor predisposing individuals to complications from sepsis; however, cardiac fibrosis was recently noted as a risk factor for severe COVID-19 (Mustroph et al., 2021), suggesting this as a potential confounding factor in our study.

In summary, our findings indicate that PAI-1 is not causally related to mortality or kidney injury during sepsis. Instead, our data suggest a potential role for PAI-1 in the resolution of sepsis and sepsis-associated AKI in the elderly. The mechanisms underlying this finding require further investigation.

## Data Availability

The raw data supporting the conclusion of this article will be made available by the authors, without undue reservation.
