# Supplementary material for: PAI-1 as a critical factor in the resolution of sepsis and acute kidney injury in old age
Source: Front Cell Dev Biol. 2024 Jan 18;11:1330433. doi: 10.3389/fcell.2023.1330433 (PMC10830627; doi:10.3389/fcell.2023.1330433)
Supplement: Supplementary file 1 [file DataSheet1.pdf]

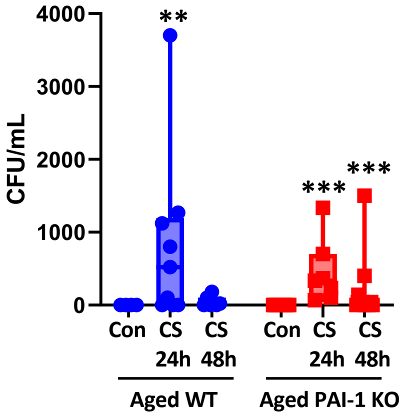

Supplementary Figure S1. Extended bacterial load data in aged mice with CS-induced sepsis. Sepsis was induced in aged (18-22 months old) WT (n=16) and PAI-1 KO (n=18) mice by cecal slurry (CS) injection. Glycerol-injected mice were used as controls. Bacteremia was assessed by anaerobic blood cultures in mice euthanized at 24 and 48h post CS-injection. Data are expressed in box plots from minimum to maximum values with a bar representing the mean, each point represents an individual mouse. Statistical difference was determined by two-way ANOVA with multiple comparisons, ** or *** indicates $p < 0.01$ or < 0.001 for control vs. each timepoint comparison. There was no significant difference between the strains.

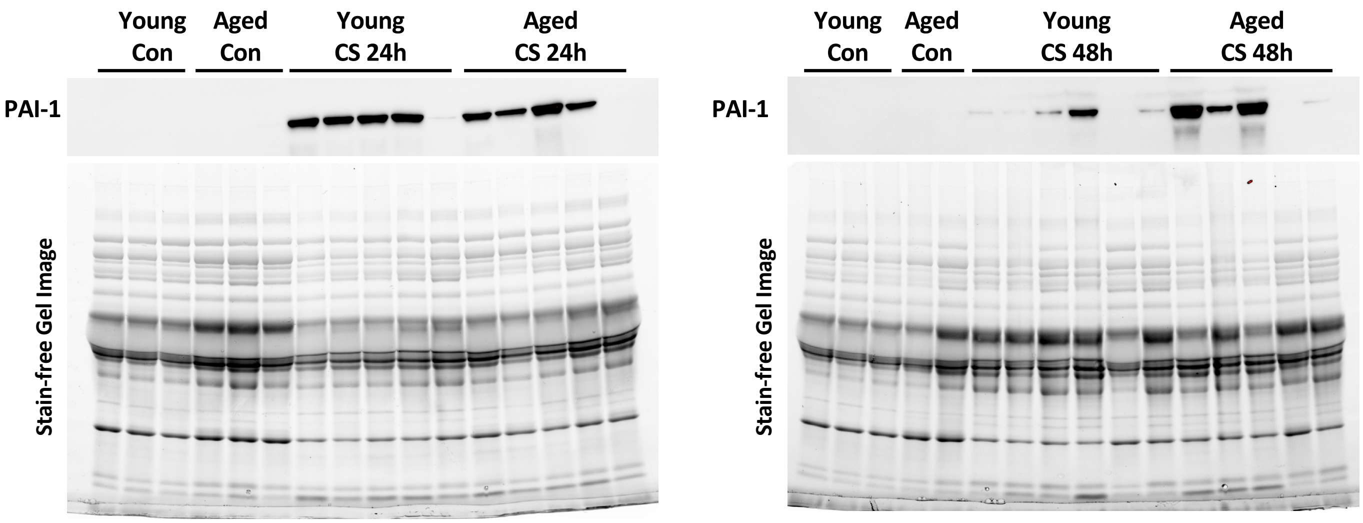

Supplementary Figure S2. Western blot images for PAI-1 quantified in Figure 3B and C. PAI-1 was assessed by western blot analyses of diluted plasma samples taken at 24 and 48h after CS-injection. Each lane represents a sample from an individual mouse. Intensity of each band was normalized for total protein content of each lane shown in the stain-free gel.

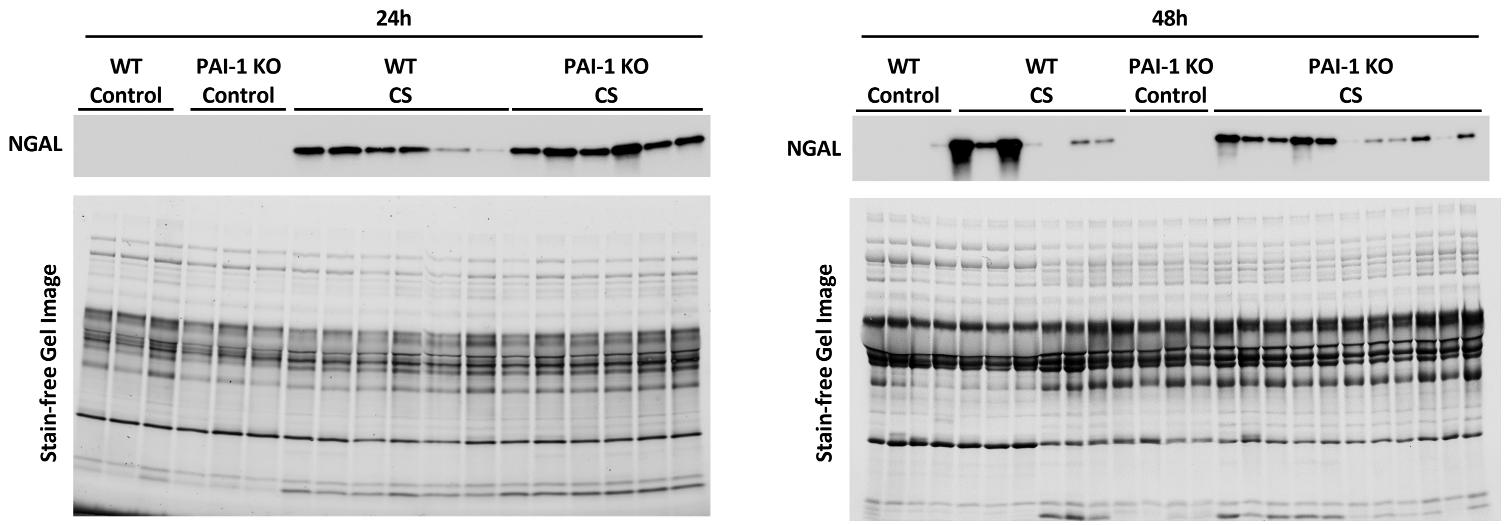

Supplementary Figure S3. Western blot images for NGAL quantified in Figure 4A. NGAL was assessed by western blot analyses of diluted plasma samples taken at 24 and 48h after CS-injection. Each lane represents a sample from an individual mouse. Intensity of each band was normalized for total protein content of each lane shown in the stain-free gel.

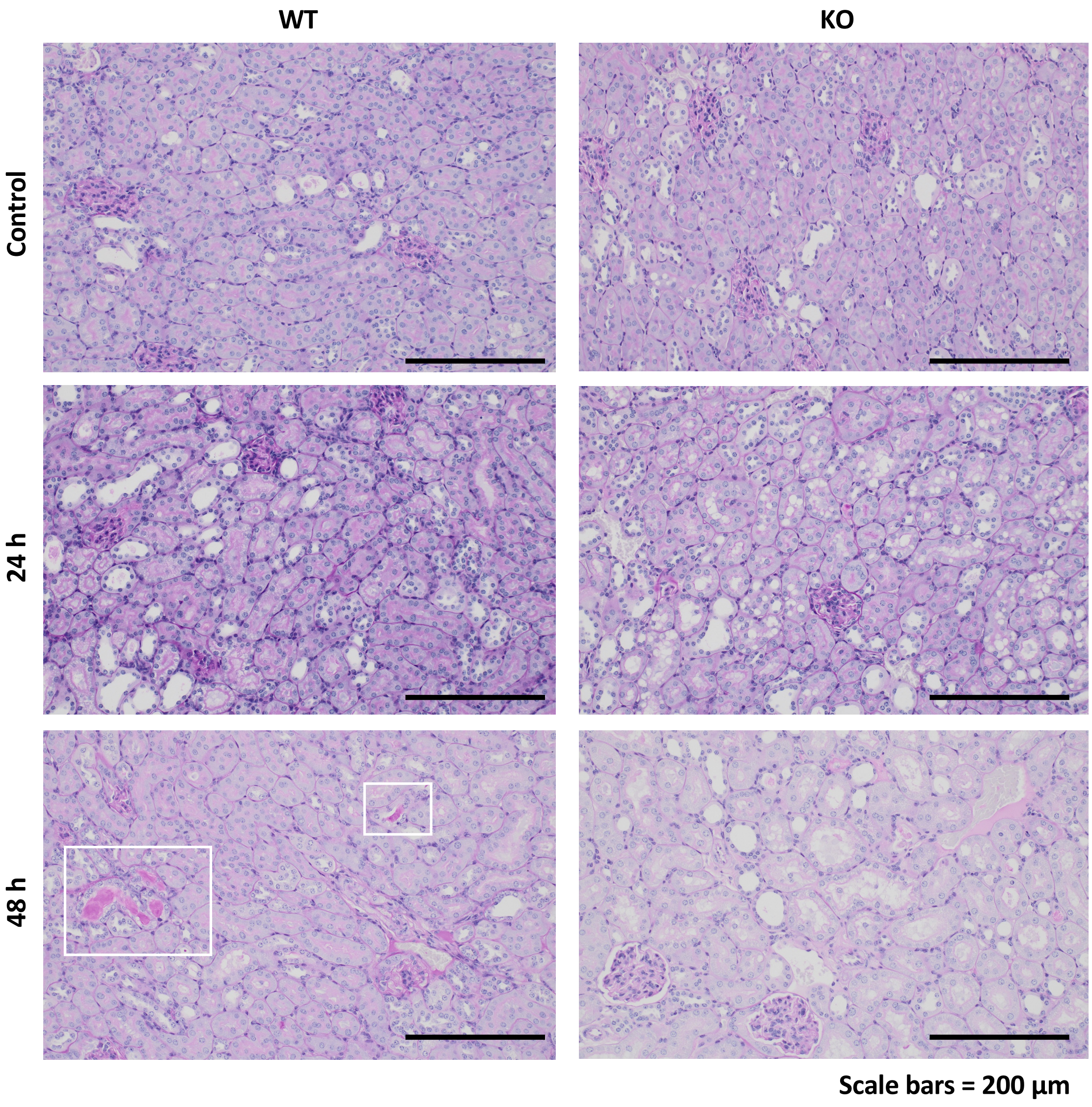

Supplementary Figure S4. Representative PAS-stained kidney sections. Tubular casts are outlined in rectangle, total magnification 10X.

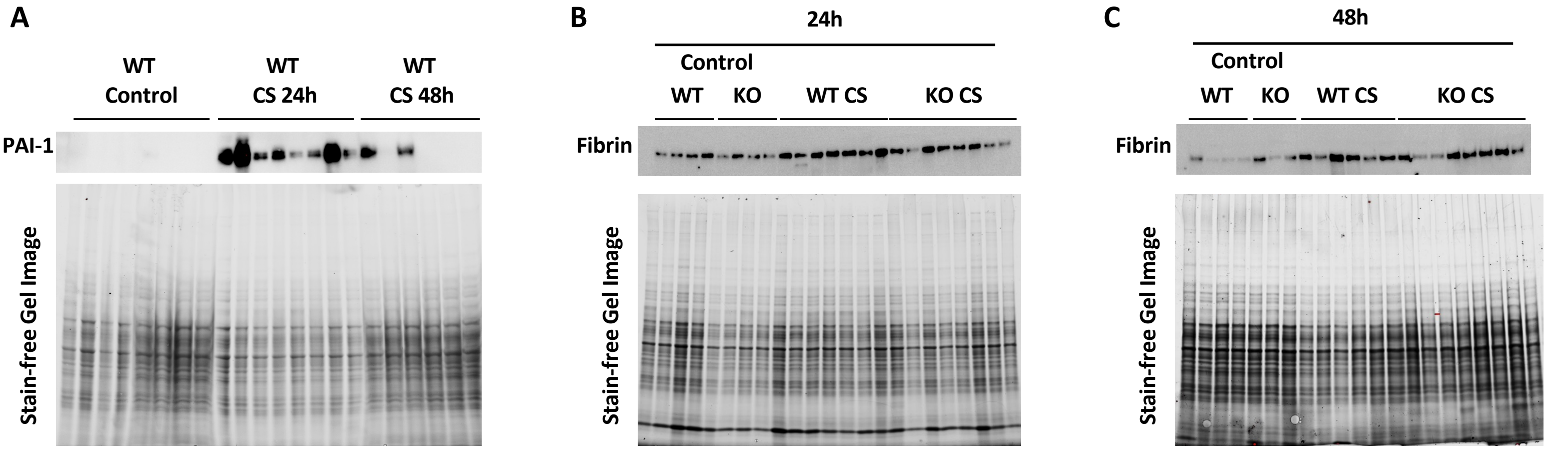

Supplementary Figure S5. Western blots and protein gel images from PAI-1 and fibrin quantification depicted in Figure 6. (A) Kidney PAI-1 protein levels in WT mice assessed by western blot. Fibrin deposition in the kidney was assessed by western blot at **(B)** 24h and **(C)** 48h. Intensity of each band was normalized for total protein content of each lane shown in the stain-free gel.

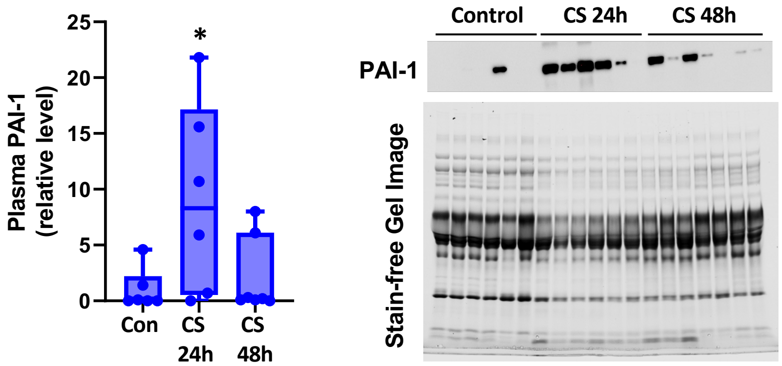

Supplementary Figure S6. Plasma PAI-1 in aged WT mice assessed by western blot. Plasma PAI-1 protein levels in WT mice assessed by western blot. Intensity of each band was normalized for total protein content of each lane shown in the stain-free gel.
